# Surgical management of groove pancreatitis: a case report

**DOI:** 10.11604/pamj.2020.36.99.21873

**Published:** 2020-06-16

**Authors:** Imen Ben Ismail, Hakim Zenaidi, Abdelwahed Yahmadi, Saber Rebii, Ayoub Zoghlami

**Affiliations:** 1 Department of general surgery, Trauma and Burns Center, Ben Arous, Tunisia

**Keywords:** Chronic pancreatitis, pancreas disease, pancreatico-duodenectomy, surgical management

## Abstract

Groove pancreatitis (GP) is a rare form of chronic pancreatitis involving the groove area bound by the pancreatic head, the duodenum, and the common bile duct. The diagnosis of this entity is challenging since it can mimic pancreatic carcinoma. We herein report the case of groove pancreatitis diagnosed in a 37 year old men, with a past history of chronic alcohol consumption. The patient was admitted for several times over the past three years because of recurrent alcohol-induced pancreatitis. The diagnosis of groove pancreatitis was made on the basis of CT, MRI and EUS findings. A medical treatment was initially attempted. In the absence of improvement in clinical symptoms, a pancreatico-duodenectomy was performed with satisfying results at 24 months follow up. Pancreatico-duodenectomy is the treatment of choice in groove pancreatitis since it leads to total resolution of clinical symptoms.

## Introduction

Groove pancreatitis (GP) is a rare form of chronic pancreatitis involving the groove area bound by the pancreatic head, the duodenum, and the common bile duct [1]. It was first described, by Becker, in 1973 [1]. GP mostly affects men in his fourth or fifth decade of life [2]. The real pathogenesis of GP has not been clearly identified. It is believed that alcohol consumption and the presence of ectopic pancreatic tissue in the duodenal wall may play a major role in its development [2]. The clinical manifestations are similar to other forms of chronic pancreatitis. Abdominal pain, vomiting, and weight loss are the main features of GP [3]. GP poses diagnostic challenges since it can mimic pancreatic carcinoma [4]. Advances in diagnostic imaging have facilitated the recognition of this condition [5,6]. Imaging typically reveals duodenal stenosis, and cystic lesions near the head of the pancreas [7]. A specific treatment strategy has yet to be established [8]. The reported treatments of GP include medical [8,9], endoscopic [10] or surgical approaches [8,9].

## Patient and observation

**Figure 1:**
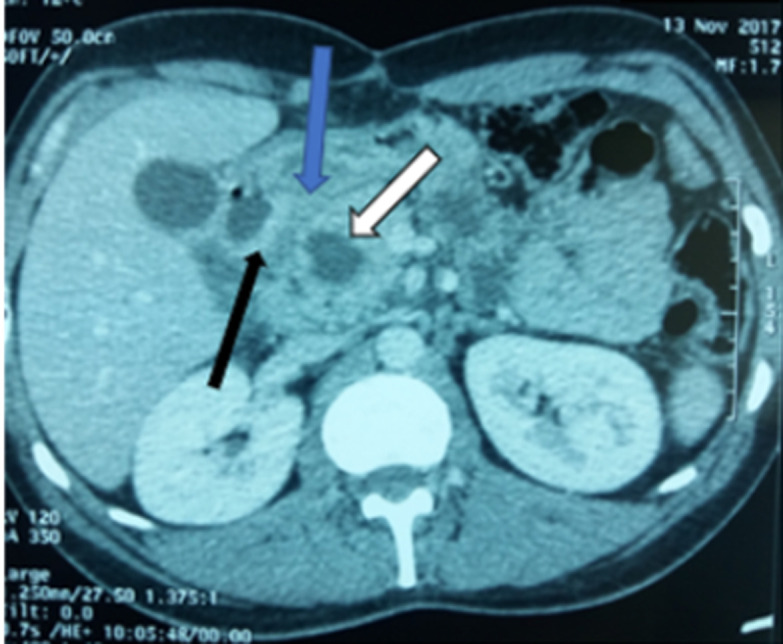
computed tomography (CT) findings: cystic lesions in the head of pancreas (white arrow), a low-density area in region of groove (blue arrow), and a hypertrophic duodenal wall (black arrow)

**Figure 2:**
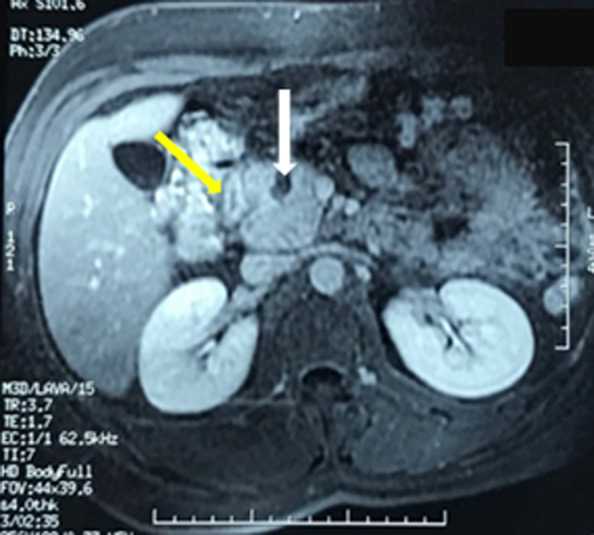
MRI findings: cystic lesions in the head of pancreas (white arrow), a hypertrophic duodenal wall (yellow arrow)

We report the case of a 37-year-old man, with a past history of chronic alcohol consumption, presented with epigastric pain radiating to the back, intermittent vomiting and recent weight loss. He had a history of recurrent admissions with abdominal pain secondary to recurrent alcohol-induced pancreatitis over the past three years. The physical examination revealed mild tenderness in the upper abdomen and periumbilical region, while no abdominal mass was detected. Biochemistry tests showed high levels of lipase and amylase (>20x normal value) and cholestasis syndrome (alkaline phosphatase and gamma glutamyl transpeptidase x 3 normal value). Tumor markers (CA 19.9 marker and carcinoembryonic antigen) were within normal limits. A CT of the abdomen was realized (Figure 1). It revealed a low density area in the pancreaticoduodenal groove, thickening of the duodenal wall and a swelling of the pancreatic head with a 15 mm hypodense cystic lesion. The common bile and pancreatic ducts appeared grossly normal. The pancreatic body and tail were normal. In order to clearly delineate the ductal system and the peri ampullary region, an MRI examination was realized (Figure 2). The later confirmed the aforementioned CT scan findings.

**Figure 3:**
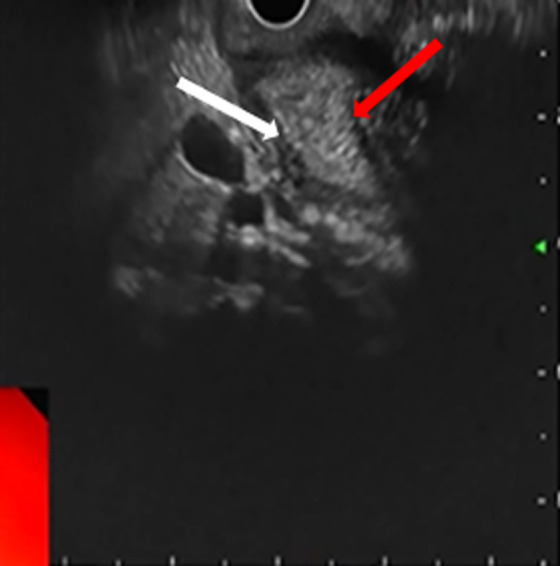
cystic lesion of the head of the pancreas (white arrow)

**Figure 4:**
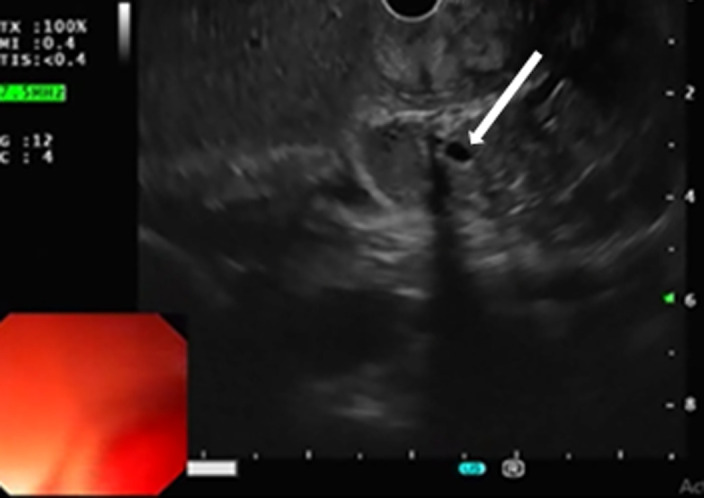
swelling of the head of the pancreas (red arrow)/ hypoechoic groove (white arrow)

Endoscopic ultrasound (EUS) was performed, showing localized wall thickening in the second portion of the duodenum. The cephalic region of the pancreas was hypoechoic, heterogeneous, with fuzzy limits. It was slightly increased in size with an areolar appearance and some parenchymal micro-calcifications. It was the seat of a 2 cm cystic formation not communicating with the Wirsung duct (Figure 3, Figure 4). These findings appeared consistent with the diagnosis of groove pancreatitis. A medical treatment with proton pump inhibitors and analgesics, was conducted, with abstinence from alcohol. Since then, the patient was suffering from recurrent abdominal pain associated with vomiting. After three months the patient developed a jaundice. Therefore, the medical management was judged to be unsuccessful and a Whipple-type pancreatico-duodenectomy was performed. The laparotomy revealed an inflammatory bulky mass in the head of the pancreas and the duodenum was adherent to the adjacent tissues. The resected specimen revealed severe fibrosis and inflammatory cell infiltration in the groove area. No evidence of malignancy was present. The post operative recovery was uneventful, with the exception of a grade A pancreatic fistula, which healed spontaneously 10 days after surgery. At 24 months´ follow-up, the patient was asymptomatic.

## Discussion

Groove pancreatitis (GP) is a rare form of chronic pancreatitis, characterised by fibrosis of the paraduodenal groove, ananatomical section limited by the pancreatic head, the duodenum and the main bile duct [1].This entity has been first described by Beckerin 1973 [1], with the German name of “Rinnen pankreatitis”, and the term of groove pancreatitis has been elaborated by Stolte *et al*. in 1982 [11]. In the early 1990s, Becker and Mischke classified GP into 2 types: the pure form, which does not involve the main pancreatic duct or the pancreatic parenchyma apart from the groove, and the segmental form, which involves the groove and the head of the pancreas with stenosis of the pancreatic duct leading to upstream dilatation [12]. Because of its rarity, the prevalence of groove pancreatitis is difficult to assess. It varies largely from 2.7 to 24.5% in surgical specimens of pancreatico-duodenectomies performed in patients with chronic pancreatitis [13]. The exact pathogenesis of GP has not been clearly identified to date, although various hypotheses have been suggested. Heavy smoking and chronic alcohol consumption are thought to be the main factors by increasing the viscosity of pancreatic juices, inducing stasis and out flow obstruction which leads to Brunner gland hyperplasia, causing occlusion or dysfunction of the minor papilla [11]. Also, the cause of this condition can be a history of gastrectomy, a gastroduodenal ulcer, biliary diseases and the presence of anatomic abnormalities which causing minor papilla dysfunction [9]. Clinical presentation resembles that of chronic pancreatitis. The patients usually present with postprandial abdominal pain and postprandial vomiting, secondary to stenosis of the duodenum, often leading to significant weight loss [14]. Blood tests might show a slight elevation of serum pancreatic however tumor markers, are seldom increased [15]. Various imaging modalities are present for the diagnosis of groove pancreatitis. CT scan often reveals a hypodense, poorly enhanced mass between the pancreatic head and a thickened duodenal wall, with cysts usually seen in the duodenal wall and/or the groove as well as duodenal stenosis due to wall thickening [5].

Magnetic resonance (MR) imaging usually presents a hypo-intense mass on T1-weighed MR images, and iso or slightly hyperintense on T2-weighed MR images, with delayed contrast enhancement after injection of the contrast material, reflecting its fibrous nature [16]. Nowadays, besides MRI, EUS is also considered a gold standard for GP diagnosis [17]. It shows smooth tubular stenosis of the common bile duct without abnormality of the main pancreatic duct. Although a lot of radiological aspects are described, distinguishing between groove pancreatitis and adenocarcinoma of the head of the pancreas is a daunting task. Recently, Kalb *et al*. have obtained an accuracy diagnosis of 87.2% for groove pancreatitis, using 3 MRI criteria: focal thickening of the duodenum 2, hyper uptake of the second segment of the duodenum, and cystic alterations in the region of the accessory pancreatic duct [18]. Treatment options of GP are categorized into conservative therapy and surgical intervention. The conservative medical measures, include the cessation of smoking and alcohol consumption, pancreatic rest, analgesics, proton pump inhibitors, pancreatic enzyme supplement and nutritional support. Endoscopic drainage of a stenotic or occluded minor duct is an important non surgical approach with good results [10]. If symptoms do not improve with the previous strategies, complications appear, or when a suspicion of a neoplasm is present, surgery must be the treatment of choice. The preferred surgical intervention is pancreatico-duodenectomy using the Whipple procedure or with preservation of the pylorus [19]. A part of the procedure used, it is proved that surgery highly improves quality of life and contributes to pain cessation in 76% of cases [2]. Recently, Chunfu and al demonstrated that GRPH (Groove Resection of Pancreatic Head): a novel surgical procedure, which involved the resection of the groove area of the pancreas only, while the duodenum, common bile duct, main pancreatic duct and the majority of the pancreatic head were preserved, is a feasible and effective technique, and this procedure may be an alternative for the surgical treatment of GP without duodenal stenosis [20].

## Conclusion

The pre operative diagnosis of groove pancreatitis is challenging. Despite the development the imaging technics, the differentiation between this entity and pancreatic head carcinoma is still difficult. Medical and endoscopic treatment may be helpful initially or in patients who are unfit for surgery, but the corner stone of therapy is pancreatico-duodenectomy since it leads to total resolution of clinical symptoms.
